# Psychosocial Adjustment of In-Home Caregivers of Family Members with Dementia and Parkinson's Disease: A Comparative Study

**DOI:** 10.1155/2020/2086834

**Published:** 2020-04-28

**Authors:** María Cristina Lopes Dos Santos, María Victoria Navarta-Sánchez, José Antonio Moler, Ignacio García-Lautre, Sagrario Anaut-Bravo, Mari Carmen Portillo

**Affiliations:** ^1^Department of Sociology and Social Work, Public University of Navarra, Pamplona, Spain; ^2^Department of Nursing. Faculty of Medicine, Autonomous University of Madrid, Madrid, Spain; ^3^Department of Statistics, Information Technology and Mathematics, Public University of Navarra, Pamplona, Spain; ^4^ARC Wessex, NIHR, School of Health Sciences, University of Southampton, Southampton, UK

## Abstract

Neurodegenerative diseases such as Parkinson's and dementia are highly prevalent worldwide. People who suffer from these disorders often receive in-home care and assistance from family members, who must dedicate a considerable amount of time to the care recipient. The study of family caregivers' psychosocial adjustment to the degenerative processes of both conditions is of interest due to the implications for the quality of life of both the care receiver and the caregiver, as well as other family members. This study compares the psychosocial adjustment of family members who care for people with dementia and Parkinson's disease and identifies the main sociodemographic variables that affect the processes of adjustment to both conditions. To this end, the Psychosocial Adjustment to Illness Scale (PAIS-SR) and a sociodemographic form were administered to 157 family caregivers in Navarre, Spain. The results show that adjustment to the disease in family caregivers of people with Parkinson's disease and dementia is, in general, satisfactory and related to variables such as place of residence, income, and employment status. The illness itself (Parkinson's or dementia), however, is found to be the most influential variable in the level of psychosocial adjustment.

## 1. Introduction

Processes of adjustment to neurodegenerative conditions in individuals and their caregivers have been the object of study in recent decades [1–3], as the manner in which both the individual and the family cope and their perceived well-being will depend on how they adjust to the new situation. Looking at specific conditions, we found empirical studies such as Navarta et al.'s. [4, 5] on Parkinson's disease, Samios et al.'s. [6] and Rintell's [7] on multiple sclerosis, Amador-Marín's and Guerra-Martín's [8] on nonpharmacological interventions to improve caregivers' quality of life, Garzón-Patterson's and Pascual-Cuesta's [1] on the psychological-behavioral symptoms of people with Alzheimer's disease and caregiver burden, Sánchez's and Fontalba's [9] on burnout in caregivers of people with dementia, and Telford et al.'s. [10] on the process of living with chronic illness.

The recent report on noncommunicable diseases published by the World Health Organization [11] recognizes that neurodegenerative diseases are one of the major health challenges worldwide, especially dementias and Parkinson's disease. Due to their enormous impact on the population, there has been increasing interest and concern about these diseases. In 2010, 35.6 million people were estimated to be affected by dementia worldwide [12], a number that is expected to increase to 131.5 million by 2050 [13]. Parkinson's is the second most common neurodegenerative disease in people over 65 and affects more than 10 million people worldwide, a figure that is also expected to grow due to population aging [14].

As for the economic burden in Western Europe, for example, the medical costs for a population with dementia of almost seven million people amounted to $30.19 billion annually, social costs to $92.88 billion annually, and informal care costs to $87.05 billion annually [12]. In the case of Parkinson's disease, the economic burden in the United States exceeded $14.4 billion per year in 2010 [15] and amounted to €13.9 billion per year in Europe [16]. Nevertheless, according to the World Health Organization [11]; the measures taken to care for people with neurodegenerative diseases have been insufficient despite this expenditure. For Australia, Dickins et al. [17] reported that 75% of people with dementia remain at home. In other cases, it has been estimated that between 60% and 80% of home care is informal [18] and that those with the disease live with their caregivers. These informal caregivers, mostly women, provide nonprofessional assistance and care to dependent persons. These studies have found that those suffering from the disease and their caregivers prefer to remain at home and highlight the importance of in-home care.

In response to dementia, the World Health Organization has underlined the need to apply health-in-all-policies and interventions, whole-of-society, and multisectoral approaches [11]. This implies that interventions that seek to be effective and socially accepted must incorporate the cultural, personal, and professional contexts of caregivers and patients [17]. In other words, the actions must be contextualized and sustainable to ensure they have an impact on the well-being and quality of life of the care receivers, while also promoting adequate training and the involvement of professionals [19]. For this purpose, the World Health Organization agreed to focus its efforts on the framework of a Global Action Plan to combat dementia [20], including the prioritization and awareness of dementia and support for dementia caregivers, research and innovation, and lower risk of dementia.

In line with this, recent research has focused on the caregiving experience of family members and the intrafamily impact of providing care in a continual and intensive manner in a neurodegenerative process associated with the loss of functions and capacities [21–25].

Among the effects on caregivers is the experiential effort in the face of uncertainties that arise as the disease progresses [5, 26]. High levels of stress and distress have been shown to exert a negative effect on the emotional state and sense of burden or overload in dementia´s [27, 28] and Parkinson's caregivers [25, 29]. More concretely, those suffering from neurodegenerative diseases, particularly Parkinson's and dementias, are the most vulnerable element of the family as a whole due to their progressive loss of functionality and roles as a result of behavioral alterations, cognitive impairment, communication deficits, and a general and progressive deterioration. The frailty of people with dementia [30, 31] and with Parkinson's [32, 33] requires caregivers to provide increasing care, for which external support at home or in institutions is sometimes needed [18, 34], thus increasing the caregivers' own frailty.

To advance in the knowledge of the impact factors and the processes of adjusting to and coping with Parkinson's disease and dementia, in this article we contribute to the literature through a comparative study of both diseases. Very few studies have focused on both diseases despite the fact that they share many aspects in common, not only in terms of the patient themselves, but also family caregivers [35]. Therefore, the objective of this study is to compare the psychosocial adjustment of family caregivers of people with dementia (PWD) and people with Parkinson's (PWP) and to determine the factors that significantly affect their processes of adjustment to both diseases.

## 2. Methods

### 2.1. Selection of Participants and Access

This study was approved by two ethics committees: the University of Navarre for Parkinson's disease-related research (cod. 020/2011) and the Public University of Navarre for dementia-related research (cod. PI-025/15).

The study participants were recruited if they met the inclusion criteria of being a constant in-home caregiver of a family member with dementia or Parkinson's disease and residing in Navarre (north of Spain). In Parkinson´s cases, caregivers were included if their relatives fulfilled the UK PD Society Brain Bank diagnostic criteria for PD according to their neurologists [36]. Caregivers of PWP who presented dementia were excluded [37]. As for the dementia cases, caregivers were included if the relatives fulfilled the ICD-10 [38] diagnostic criteria for dementia.

In both cases, information on health status of caregivers was collected, resulting in 57.8% of PWP and 33% of their family caregivers reported having a disease. These diseases were mainly arterial hypertension and osteoarthritis.

Professionals from 19 municipal social service agencies located throughout Navarre, three primary care health centers, Alzheimer's (AFAN) and Parkinson's (ANAPAR) associations, and a hospital of Pamplona (for Parkinson's cases) collaborated in the recruitment process.

Caregivers interested in participating were contacted by the research team to provide them further information about the study and requested that they sign informed consent. In general, the interviews were conducted in the participants' households and lasted an average of one hour per interview.

Data on Parkinson's cases were collected from June 2013 to December 2013 and from February 2015 to October 2016 for cases of dementia. Participants were recruited through nonprobability convenience sampling [39]. A total of 157 family caregivers participated: 74 PWD caregivers and 83 PWP caregivers.

### 2.2. Data Collection and Instruments

Data were collected by means of a sociodemographic data form and the PAIS-SR, which is the self-report version of the Psychosocial Adjustment to Illness Scale (PAIS) of Morrow et al. [40] developed by Derogatis [41]. The PAIS-SR comprises 46 items grouped into seven dimensions: healthcare orientation, vocational environment, domestic environment, sexual relationships, extended family relationships, social environment, and psychological distress. Each item is scored on a 4-point Likert scale measured from 3 to 0 points on even questions and 0 to 3 points on odd questions [42]. The PAIS-SR questionnaire items were recoded: a score of 0 indicates that the caregiver evaluates the item positively (very satisfied), while a score of 3 indicates that the caregiver evaluates the item negatively (very dissatisfied).

The scale, therefore, allows results to be obtained at both the dimension level and at the specific item level [41]. The global score indicates the respondents' general adjustment to the disease, while the dimension or item score evaluates specific areas or aspects of psychosocial adjustment. A total score of more than 62 points indicates that the respondent has adjusted to the disease, while a score below 62 indicates that the respondent has not adjusted to the disease [42].

The caregiver/family version of PAIS-SR is not validated in Spanish. Therefore, permission was requested from the authors to use the scale and a back-translation process was carried out [43] by three bilingual experts in psychosocial research and neurological diseases. The Spanish version used here was obtained via this back-translation process.

In accordance with the global scoring criteria of Derogatis and Derogatis [42], a categorical variable of satisfaction (good/poor) called “adjustment” was constructed. The lack of responses from PWD caregivers (67 out of 74 participants) in the “professional” and “sexual relations” dimensions required a modification: summing the remaining sections (five in total) and the classification of a global score below 45 as “good” or satisfaction and a score above 45 as “poor” or dissatisfaction. To validate this modification, all participants for whom the variable “adjustment” could be obtained without modification were considered, and the number of individuals who were misclassified when applying the modification was counted. The result was only two incorrectly classified individuals, thus indicating that the procedure to modify the scale was valid.

### 2.3. Data Analysis

The statistical analysis was performed using Software R together with the integrated package Factor MineR [44] and SPAD8 [45]. First, the existence of association (*p* < 0.05) between the responses to each item and the illness of the care receiver was determined. In a second step, in order to establish the sociodemographic profile according to the “adjustment” variable of the caregiver, two complementary techniques were used.

Firstly, the simple correspondence analysis (CA) of Lebart, Morneau, and Piron [46] was performed, which provided an initial set of variables that influence the variable adjustment. With this initial set of explanatory variables, a logistic regression fit was used. The significant variables in this regression (*p* < 0.05) provided the caregivers' profile according to their adjustment to the illness.

Secondly, a cluster analysis was carried out to group the respondents according to the scale responses as a whole. This analysis is based on the multiple correspondence analysis (MCA) of Lebart et al. [46]. All the information was extracted from the PAIS-SR scale, with the exception of dimensions II and IV due to the high nonresponse rate regarding these dimensions, and each question was considered an original variable. Two artificial variables (factors) were created that retain, to the greatest extent possible, the initial information. Thus, respondents are represented according to the value assigned to them in these two factors.

## 3. Results

Of the total sample of 157 family caregivers, 76.4% were married, half of whom were the daughter or son of the care receiver (Table 1). Only 35.7% of the caregivers worked and, of those who did not, most were retired. The majority lived in urban areas and only a few in rural areas. The standard deviation (SD) of years of care was 6.7.

The profile of the caregiver was that of a woman (77.70%), mostly the daughter (45.86%) or wife (41.40%) of the care receiver, with a mean age of 60.5 years. However, depending on whether the illness was Parkinson's or dementia, some differences in the caregiver's profile have been found in terms of kinship (predominance of the care receiver's children in dementia´s cases: 70.3%), retirement status (mostly Parkinson's cases), and place of residence (urban in Parkinson's cases and semirural in dementia´s cases).

### 3.1. Psychosocial Adjustment of Caregivers by PAIS-SR Dimensions

The assessment of information received about the disease and treatment revealed differences in attitudes towards healthcare (dimension I) depending on whether the respondent was a PWP caregiver (more than 50% negative) or a PWD caregiver (more than 70% positive). In the rest of the items, the distribution was similar for both cases (Table 2). At least 80% of the caregivers surveyed indicated they were satisfied with the medical care and assistance received and over 90% stated that they had no hope the care receiver would recover.

As regards the situation in the household (dimension III), the level of satisfaction was good (85% of the total caregivers) when measuring the caregiver's relations with the care receiver and the rest of the cohabitants in the household (Table 3). With regard to the effect on communication, the evaluation was generally positive but was very positive in the case of PWP caregivers (above 90%).

Caregivers' assessment of the impact on their work and domestic duties showed the opposite sign and was more pronounced in the case of PWD caregivers. Regarding the need for help and help received from the rest of the family, 80% of the PWP caregivers stated they were satisfied, while approximately 60% of the PWD caregivers said they were dissatisfied (Table 3).

The influence of care on health status was generally low, although it was higher among PWP caregivers. The difference was more pronounced regarding economic status, as no PWP caregivers were dissatisfied with their economic situation compared to 40.6% of PWD family caregivers who stated they were.

As regards the rest of the dimensions (V, VI, and VII) of the PAIS-SR, the *p* value indicated few differences between family caregivers of PWP and PWD. Very slight differences were found regarding the relationship with noncohabitant family members (dimension V), since 80% of PWP caregivers stated they were satisfied and slightly more than half of the responses of PWD caregivers were positive. It is also interesting to note the dissatisfaction of PWD caregivers (67%) regarding noncohabiting family members.

In terms of leisure and free time (dimension VI), PWP caregivers showed an interest in leisure activities (above 80%), although their satisfaction with the actual participation in these activities was lower (around 50%). The same behavior was observed among PWD caregivers, but the percentages of satisfied respondents were lower (50% for interest and 25% for actual participation). According to the MCA, 20% of the total sample was interested and effectively engaged in leisure activities (the majority of PWP caregivers). A similar percentage was neither interested nor engaged in leisure activities, particularly PWD caregivers.

Finally, as regards the evaluation of the caregivers' psychological status (dimension VII), no significant difference was found between the groups, with the exception of self-blame. In this case, those caring for PWD showed a greater tendency towards affirmative responses (dissatisfaction).

### 3.2. Global Profile of the Adjustment Variable

In order to define the global profile of the sociodemographic variables that significantly influenced psychosocial adjustment to the disease, three analyses were performed. The first general result indicated that 63.5% of PWD caregivers could be classified as good (positive adjustment), while this percentage increased to 95.2% among PWP caregivers.

**Figure 1:**
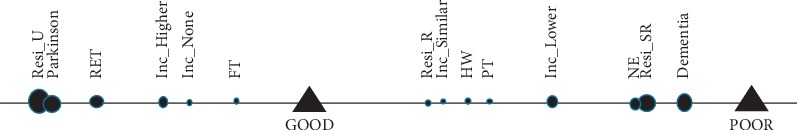
Projection of correspondence analysis. ^*∗*^Resi_U, urban residence; RET, retired; Inc_Higher, above average income; Inc_None, no income; FT, full-time job; Resi_R, rural residence; Inc_Similar, income similar to average; HW, housewife; PT, part-time job; Inc_Lower, lower-than-average income; NE, nonemployed; Resi_SR, semirural residence.

As regards the prospective study, the analysis of simple correspondences with all the sociodemographic variables showed that the categories most associated with the two levels of adjustment (good/poor) were type of disease, followed by place of residence, employment status, and income. The categories of these four variables with respect to the two adjustment levels are shown in Figure 1.

Significantly, the categories referring to caregivers of PWP are located to the left of the GOOD point, which means that these caregivers had adjusted well to Parkinson's disease. For caregivers of PWD, none of the categories are to the right of the reference point POOR (no category has more than 50% of these individuals), suggesting that there was a tendency to negative adjustment, but without a generalized maladjustment.

Table 4 shows the profile of sociodemographic variables using two techniques. On the one hand, we considered variables in which the association was significant (the chi-square test). The result showed that caregivers of PWP who lived in urban areas and had an above average income were the best adjusted.

On the other hand, the logistic regression (dependent variable, good adjustment; explanatory variables, sociodemographic variables) showed that income did not have a significant influence on adjustment. Moreover, PWP caregivers were nine times more likely to adjust well to the situation than PWD caregivers, while living in a nonurban area reduced the likelihood of good adjustment by one sixth.

These three analyses indicated that the main variables of psychosocial adjustment of PWP and PWD caregivers were related to the type of disease (Parkinson's or dementia) and place of residence (urban, semiurban, and rural). To better visualize the profiles of PWP and PWD caregivers and their relationship with these variables, a cluster analysis was also performed.

**Figure 2:**
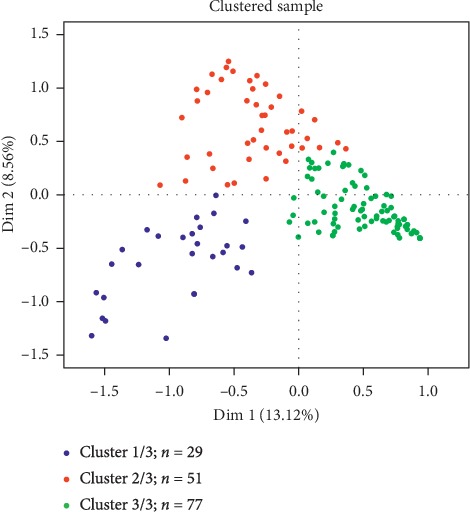
Cluster analysis according to the responses of family caregivers of PWP and PWD.

The results revealed three situations of psychosocial adjustment to the disease by family caregivers as follows (see Figure 2):

- Cluster 1/3 included 29 family caregivers with a global score of 3 for the items on the PAIS-SR scale, thus indicating a negative adjustment to changes resulting from the disease. Approximately 80% of the respondents in this group were PWD caregivers, of which more than 60% adjusted poorly to the disease, had “other income,” and lived in semirural areas.
- Cluster 2/3 was comprised of 51 family caregivers who mostly obtained a score of 2 for the items on the PAIS-SR scale. Their degree of adjustment was medium and low. About 70% of those in this group were PWD caregivers, of which more than 80% were under 70 years of age, more than 60% coped poorly with the disease, 60% were the daughter or the son of the care receiver, and slightly over 50% lived in semirural areas.
- Cluster 3/3 included 77 caregivers with a score of 0 for the items on the PAIS-SR scale. Their psychosocial adjustment to the changes resulting from the disease was good or positive. A total of 80% were family caregivers of PWP. Of these, almost 75% had no other income, 60% lived in urban areas, 43% had a very high income, and 31% were retired.

## 4. Discussion

This study provides comparative results of the psychosocial adjustment of in-home caregivers of family members with Parkinson's disease and dementia. In general terms, the psychosocial adjustment of these caregivers to the illnesses of their family members (PWP and PWD) has been found to be satisfactory or good.

Several studies have highlighted the psychosocial impact of these illnesses on the closest caregivers. On some occasions, caregivers' full-time dedication to providing intensive care due to the frailty of their ill family members [31, 32] leads to a sense of burden and loss of quality of life [1, 25]. Other studies place more emphasis on the confluence of different psychosocial factors to explain how burden hinders adjustment and hence makes coping with the disease difficult [4, 26]. The research, such as that cited above, has focused more on the negative consequences of in-home care for family caregivers. Without questioning this impact, this study has identified a very different result, as the overall assessment of the psychosocial adjustment of family caregivers of PWP and PWD has been good. However, this psychosocial adjustment is more vulnerable among PWD than PWP caregivers. The difference in the cognitive impairment between the PWD and PWP could explain this result [47], as the number of years of care and mean age of the caregivers did not vary in the sample. Caregivers of PWP with dementia have reported the worst quality of life, compared with caregivers of PWP with normal cognition or mild cognition [47], and in the present study, PWP did not present dementia.

The degeneration of cognitive and communicative functions in PWD significantly affects the communication skills of the care receiver and hinders interaction with the caregiver. In addition to this effect on communication, there is a greater impact on working life and household duties than for those who care for PWP. This result could be due to the fact that, in our sample, mostly daughters or sons cared for parents with dementia, while couples mostly cared for PWP. In addition, 42% of those caring for family members with dementia worked compared to 30% of those caring for PWP. This result may explain why the illness had a greater negative impact on the daily lives of family caregivers of PWD than those who cared for PWP.

Given that three quarters of PWP and PWD caregivers were married in our sample, the rest of the family was also be affected. Cameron and Moss [21], Brodaty and Donkin [48], and Tartaglini, Ofman, and Stefani [27] reported the significant impact of in-home care on families. The participants in this study have also showed this impact. In general terms, they considered both relationships with their partner and with other cohabitants to be positive. Nevertheless, the results in this regard were less positive among those who cared for PWD and, of these, especially the partner of the daughter or son who was the caregiver. Therefore, an effect on the family and other relatives has been found, although it is less negative than reported in previous studies.

In addition to how such situations affect the nuclear family (both the caregiver and other members), this research has identified three further variables which influence the adjustment process of family caregivers of people suffering from Parkinson's disease or dementia: employment status, income, and place of residence. This result is in line with studies such as that of Dickins et al. [17] on the risk of dementia care. According to the authors, the personal context (cultural and professional) must be taken into account to identify and understand different perspectives and discourses on a social reality such as care. In our case, socioeconomic and, above all, geographical contexts form part of this personal context.

As regards less specific variables, the logistical regression has shown that two variables influenced adjustment to the disease after omitting the caregiver's employment status. Thus, the best psychosocial adjustment was found in PWP caregivers who live in urban areas and have an above average income. On the other hand, PWD family caregivers with a medium or low income living in rural and semirural areas experienced the worst psychosocial adjustment.

Moreover, the logistic regression has allowed us to further verify the factors that most influenced the psychosocial adjustment of family caregivers. These included the type of disease (Parkinson's disease or dementia) and the place of residence. In other words, the social and geographical context is found to be relevant although it is not always valued in its proper measure. Understanding the importance of this context is a key to understanding discourses regarding the discrimination of social and health policies towards people living in rural and semirural environments, as well as the need for universal accessibility to services of all kinds and economic aid that can improve citizens' welfare [49].

These results are not surprising in a general context of urbanization, globalization, and the maximum effectiveness and efficiency of actions [50]: populations are concentrated in urban areas, as are most cases of poverty, marginalization, and certain diseases [51]. Hence, it is precise that these areas provide public and private services of all kinds, as well as better means to access them, while the farther away people are from these centers, the fewer services, and less accessibility available to them.

The invisibility of nonurban areas in our study due to the large number of cases used as a central variable should not be a reason to not intervene. The fact that we have identified certain geographical areas with worse adjustment highlights the need to develop specific social-health intervention programs for these disadvantaged populations and areas within the framework of public policies.

In this same vein, previous research such as Lageman et al.'s [52], and Losada et al.'s [24] has shown the need to intervene on the biopsychosocial consequences of informal care. In both Parkinson's disease and dementia, caregivers play an important role in helping patients manage their disease and need support to care for their family members [52]. Multicomponent and psychoeducational interventions focused on improving caregivers' skills for dealing with symptoms, treatment, and coping with social changes can facilitate the development of the caregiver role [52]. These interventions should be provided to caregivers of PWD and PWP because there are similarities in the symptoms they have to learn such as visual hallucinations or emotion recognition deficits [53, 54] and also in the doubts and worries they find as caregivers of a person with a chronic disease [55]. This research, however, underlines the lack of applicable, comprehensive proposals, even at the institutional level [35]. Indeed, everything seems to suggest that it is the families themselves that must seek solutions by their own means to the situations that arise.

One aspect to take into account in this reflection is the assessment of the quality of healthcare (information received, medical care, and treatment by health professionals), which, in the case of Spain, is universal and free and therefore has a broader level of accessibility than other countries.

In terms of average satisfaction with the information received about the disease and its treatment, family caregivers of PWP and PWD coincide. However, the reports of PWP caregivers are less favorable in this regard. Greater satisfaction (high average) was found for medical care and professional treatment, with few differences between the types of disease. This is a very important aspect of the work carried out by professional teams in community healthcare centers, who engage in close and trusting relationships for an extended period of time during their patients' lives, since they are also present in semirural and rural areas.

Finally, it is important to mention the limitations of the contributions of this research. Firstly, the difficulties involved in recruiting a representative sample meant that the study was confined to only to one region of northern Spain: Navarre. Access to family caregivers was possible thanks to knowledge of the networks of health and social service professionals, actual relationships with people with Parkinson's disease and dementia, and their caregivers, as well as the respective patients' associations. The fact that the population, care services, and associations are concentrated in urban areas has led to an overrepresentation of participants from urban areas. Likewise, the fact that the stage of the disease of the PWP and PWD was not included can be understood as a further limitation. However, this factor was ruled out from the beginning because our aim was not to seek a cause-effect relationship between the stages of the diseases and psychosocial impacts on processes of adjustment.

Finally, it has been detected that a 5% of the total sample of family caregivers presented some mental health problem (anxiety or depression), which should be taken into account when nuancing the result analysis.

## 5. Conclusions

This article has explored the reality of caring for PWP and PWD in relation to the psychosocial adjustment of family caregivers. It is important to note that, despite the differences between the processes of Parkinson's disease and dementia and the different manifestations of each of these ailments, family caregivers stated that their psychosocial adjustment to the disease of PWP and PWD was good.

Likewise, the main variables influencing good psychosocial adjustment to the illness by family caregivers of PWP and PWD have been identified, although socio-health interventions must recognize the specific nature of each case in order to adapt the central variables of both neurodegenerative diseases. In doing so, it will be possible to respond more effectively to the continual changes that impact directly on family caregivers as these diseases progress.

## Figures and Tables

**Table 1:** Sociodemographic characteristics of the sample of family caregivers of PWD and PWP.

Sociodemographic characteristics	Total sample (*n* = 157)	Family members of PWD (*n* = 74)	Family members of PWP (*n* = 83)
Gender			
Female	122 (77.7%)	58 (78.3%)	65 (78.3%)
Age	60.5 years S.D.^*∗*^ = 13.19	58 years S.D.^*∗*^ = 12.5	63 years S.D.^*∗*^ = 13.9
Relationship			
Son/daughter	79 (50.3%)	52 (70.3%)	27 (32.5%)
Husband/wife	61 (38.8%)	19 (26.3%)	42 (50.6%)
Others	17 (10.8%)	4 (5.4%)	14 (16.9%)
Marital status			
Married	120 (76.4%)	54 (72.9%)	66 (79.5%)
Years as caregiver	6.7 years S.D.^*∗*^ = 6.5	6.6 years S.D.^*∗*^ = 5.1	6.9 years S.D.^*∗*^ = 7.6
Employment status			
Housewife	39 (24.8%)	18 (24.3%)	21 (25.3%)
Part-time job	40 (25.5%)	21 (28.4%)	19 (22.9%)
Full-time job	16 (10.2%)	10 (13.5%)	6 (7.2%)
Retired	46 (29.3%)	17 (22.9%)	29 (34.9%)
Nonemployed	16 (10.2%)	8 (10.8%)	8 (9.6%)
Place of residence^*∗∗*^			
Urban	68 (43.3%)	16 (21.3%)	52 (62.7%)
Semirural	67 (42.7%)	52 (69.3%)	15 (18.1%)
Rural	23 (14.6%)	7 (9.3%)	16 (19.3%)

^*∗*^S.D., standard deviation. ^*∗∗*^Urban, >10,000 inhabitants; semiurban, 3000–10,000 inhabitants; rural, <3000 inhabitants.

**Table 2:** Information from dimension I of the PAIS-SR.

Items	Satisfaction (%)				*χ* ^2^ test for association
	0	1	2	3	*p* value
On the disease and treatment					
Information about the disease					<0.00001
Caregivers of PWD	39.2	33.7	8.1	5.4	
Caregivers of PWP	33.7	15.7	19.3	31.3	
Information about the treatment					0.0004
Caregivers of PWD	54.1	21.6	20.3	4.1	
Caregivers of PWP	33.7	13.3	26.5	25.5	

On the quality of healthcare					
Quality of medical care					0.3133
Caregivers of PWD	39.2	50.0	5.4	5.4	
Caregivers of PWP	49.4	37.3	9.6	3.6	
Treatment by healthcare staff					0.8926
Caregivers of PWD	41.9	40.5	10.8	6.8	
Caregivers of PWP	44.6	37.3	13.3	4.8	
Treatment expectations					0.4885
Caregivers of PWD	56.2	35.6	2.7	5.5	
Caregivers of PWP	61.4	30.1	6.0	2.4	

0, very satisfied; 1, satisfied; 2, dissatisfied; 3, very dissatisfied.

**Table 3:** Information from dimension III of the PAIS-SR.

Items	Satisfaction (%)				*χ* ^2^ test for association
	0	1	2	3	*p* value
General relations with partner and other cohabitants					
Relationship with partner					0.07
Caregivers of PWD	58.3	27.8	9.7	4.2	
Caregivers of PWP	74.7	20.5	4.8	0.0	
Relationship with other cohabitants					0.24
Caregivers of PWD	63.6	25.8	6.1	4.5	
Caregivers of PWP	75.9	21.7	1.2	1.2	
Communication with partner and other cohabitants					<0.00001
Caregivers of PWD	41.9	28.4	12.2	17.6	
Caregivers of PWP	85.5	9.6	3.6	1.2	

Work and domestic duties					
Influence of disease on work and domestic duties					<0.00001
Caregivers of PWD	0.0	40.5	29.7	29.7	
Caregivers of PWP	43.4	38.6	13.3	4.8	
Problems with household chores					<0.00001
Caregivers of PWD	8.1	27.0	39.2	25.7	
Caregivers of PWP	74.7	10.8	7.2	7.2	
Need help					<0.00001
Caregivers of PWD	23.0	18.9	37.8	20.3	
Caregivers of PWP	67.5	13.3	12.0	7.2	

Economic and health status					
New physical illness					0.005
Caregivers of PWD	44.6	14.9	20.3	20.3	
Caregivers of PWP	50.6	27.7	18.1	7.2	
Economic difficulties					<0.00001
Caregivers of WD	28.8	42.5	23.3	2.4	
Caregivers of PWP	90.4	4.8	2.4	2.4	

0, very satisfied; 1, satisfied; 2, dissatisfied; 3, very dissatisfied.

**Table 4:** Psychosocial adjustment according to most significant sociodemographic variables.

Sociodemographic variable	Adjustment (%)		*χ* ^2^ test for association	Logistic regression (1)
	Good	Poor	*p* value	Exp (Cf) *p* value
Disease			^*∗∗∗*^	
Dementia	37.3	87.1		
Parkinson	62.7	12.9		9.12^*∗∗∗*^

Place of residence			^*∗∗∗*^	
Urban	51.6	9.7		
Rural	14.3	16.1		0.16^*∗*^
Semirural	34.1	74.2		0.17^*∗∗*^

Income			^*∗*^	Not significant
Lower than average	21.4	35.5		
Average	16.7	19.4		
Above average	38.1	16.1		
No income	13.5	6.5		
Unknown	10.3	22.6		

^*∗∗∗*^ *p* value <0.005; ^*∗∗*^*p* value <0.01; ^*∗*^*p* value <0.05; (1) dependent variable; adjustment; 1, good; 0, poor; reference categories, dementia-urban Exp (Coef) = 4.69 (^*∗∗*^); Exp(Cf) indicates the effect of a category in the adjustment odd ratio.

## Data Availability

The data used to support the findings of this study are available from the corresponding author upon request.
